# Thermal effects of thulium: YAG laser treatment of the prostate—an in vitro study

**DOI:** 10.1007/s00345-021-03805-3

**Published:** 2021-09-02

**Authors:** Simon Hein, Ralf Petzold, Rodrigo Suarez-Ibarrola, Martin Schoenthaler, Christian Gratzke, Arkadiusz Miernik

**Affiliations:** Department of Urology, Division of Urotechnology, Medical Centre, Faculty of Medicine, University of Freiburg, Hugstetter Str. 55, D-79106 Freiburg, Germany

**Keywords:** Thulium:YAG, Thermal effect, Tm:YAG laser enucleation (ThuLEP), Tm:YAG laser vaporization (ThuVEP)

## Abstract

**Purpose:**

To objectively determine whether there is potential thermal tissue damage during Tm:YAG laser-based LUTS treatment.

**Methods:**

Our experimental model was comprised of a prostatic resection trainer placed in a 37 °C water bath. In a hollowed-out central area simulating the urethral lumen, we placed a RigiFib 800 fibre, irrigation inflow regulated with a digital pump, and a type K thermocouple. A second thermocouple was inserted 0.5/1 cm adjacently and protected with an aluminum barrier to prevent it from urethral fluid. We investigated continuous and intermittent 120 W and 80 W laser application with various irrigation rates in eight measurement sessions lasting up to 14 min. Thermal measurements were recorded continuously and in real-time using MatLab. All experiments were repeated five times to balance out variations.

**Results:**

Continuous laser application at 120 W and 125 ml/min caused a urethral ∆T of ~ 15 K and a parenchymal temperature increase of up to 7 K. With 50 ml/min irrigation, a urethral and parenchymal ∆T of 30 K and 15 K were reached, respectively. Subsequently and in absence of laser application, prostatic parenchyma needed over 16 min to reach baseline body temperature. At 80 W lower temperature increases were reached compared to similar irrigation but higher power.

**Conclusions:**

We showed that potentially harming temperatures can be reached, especially during high laser power and low irrigation. The heat generation can also be conveyed to the prostate parenchyma and deeper structures, potentially affecting the neurovascular bundles. Further clinical studies with intracorporal temperature measurement are necessary to further investigate this potentially harming surgical adverse effect.

## Introduction

The Thulium:yttrium–aluminium-garnet laser (Tm:YAG) offers several techniques for the treatment of male lower urinary tract symptoms (LUTS). The main modalities are enucleation (ThuLEP), vapoenucleation (ThuVEP), vaporisation (ThuVAP), and vaporesection (ThuVARP) of the prostate [1]. Current EAU guidelines provide the following recommendations regarding the use of Tm:YAG lasers in LUTS treatment: (1) Offer ThuVEP and ThuLEP to men with moderate to severe LUTS as alternatives to transurethral resection of the prostate (TURP) and Holmium laser enucleation (HoLEP), weak recommendation. (2) Offer ThuVEP to patients under anticoagulant therapy, weak recommendation. (3) Offer ThuVARP as an alternative to TURP, strong recommendation. (4) Offer ThuVARP to patients under anticoagulant therapy, weak recommendation. [2]. Furthermore, the EAU guidelines endorse laser techniques as an alternative treatment to TURP for prostate volumes 30-80 ml and as an alternative treatment to open prostatectomy / HoLEP / bipolar enucleation for volumes > 80 ml [2].

Symptom relief efficacy with regard to IPSS, Qmax and PVR of all aforementioned Tm:YAG techniques has been shown in two meta-analyses (ThuVARP vs. TURP, [3, 4]), several ThuVEP case series [5, 6], and two RCTs comparing ThuLEP vs. bipolar enucleation/ bTURP [7, 8].

Overall, Tm:YAG techniques are characterized by a low perioperative morbidity and high safety as shown by Gross et al. in a case series with 1080 ThuVEP procedures [9]. Long-term outcomes after 12-month follow-up showed no significant IIEF-5 alterations when comparing TURP and ThuLEP in a RCT in which pre- (~ 20) and postoperative (~ 21) IIEF-5 scores were evaluated [10]. Furthermore, the same study revealed no iatrogenic stress incontinence in the ThuLEP group, while in the TURP group only one patient (2.1%) resulted with postoperative stress incontinence [10]. However, in both groups a significant intermittent postoperative urge incontinence (23.1% vs. 31.3% in TURP) was detected [10]. The pathological mechanisms of atypical postoperative complications after transurethral LUTS treatment and transient urge incontinence—independently from the source of energy—are not entirely understood. In several recent in vitro and animal studies, significant temperature increases of the irrigation fluid were confirmed in both Ho:YAG- and Tm:YAG models [11–16]. A theoretically available laser power up to 200 W in ThuVA(R)P, besides a high tissue ablation efficiency, might bear a high risk for the development of significant temperature increases of the surrounding irrigation fluid and tissue.

In the present study, we evaluate the intraparenchymal and urethral thermal effects of a Tm:YAG laser in a standardized in vitro setting. The study aims to determine if thermal damage exists to surrounding structures during Tm:YAG laser-based LUTS treatment.

## Materials and methods

**Fig. 1:**
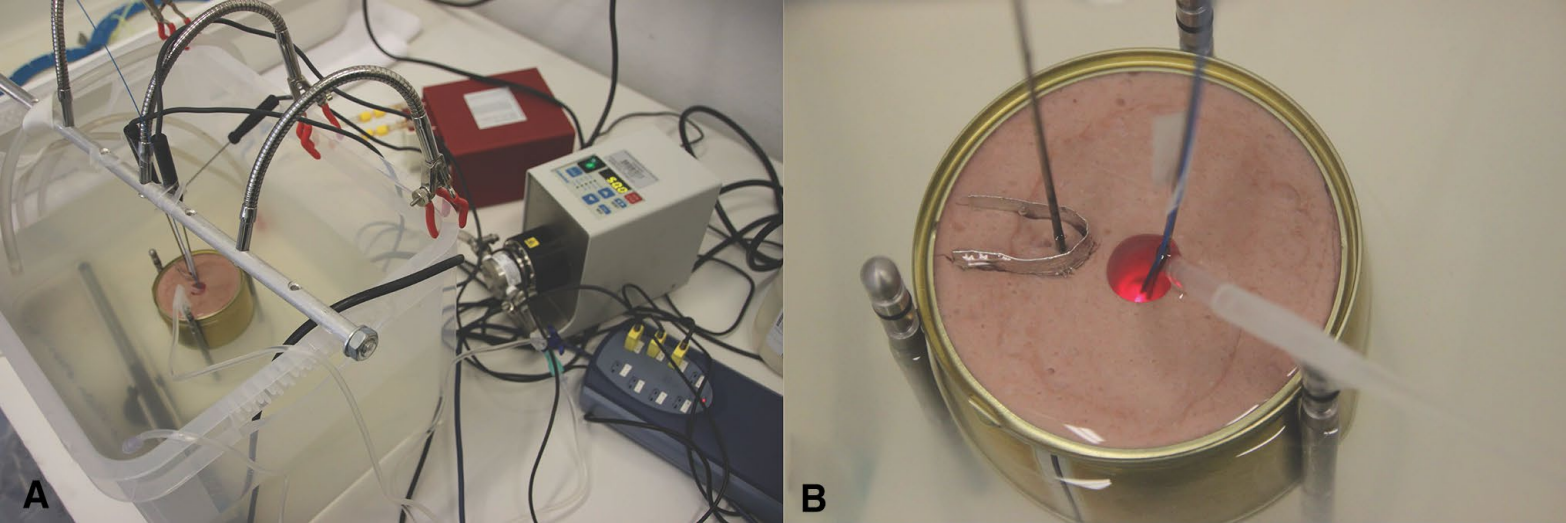
Experimental setup

**Fig. 2:**
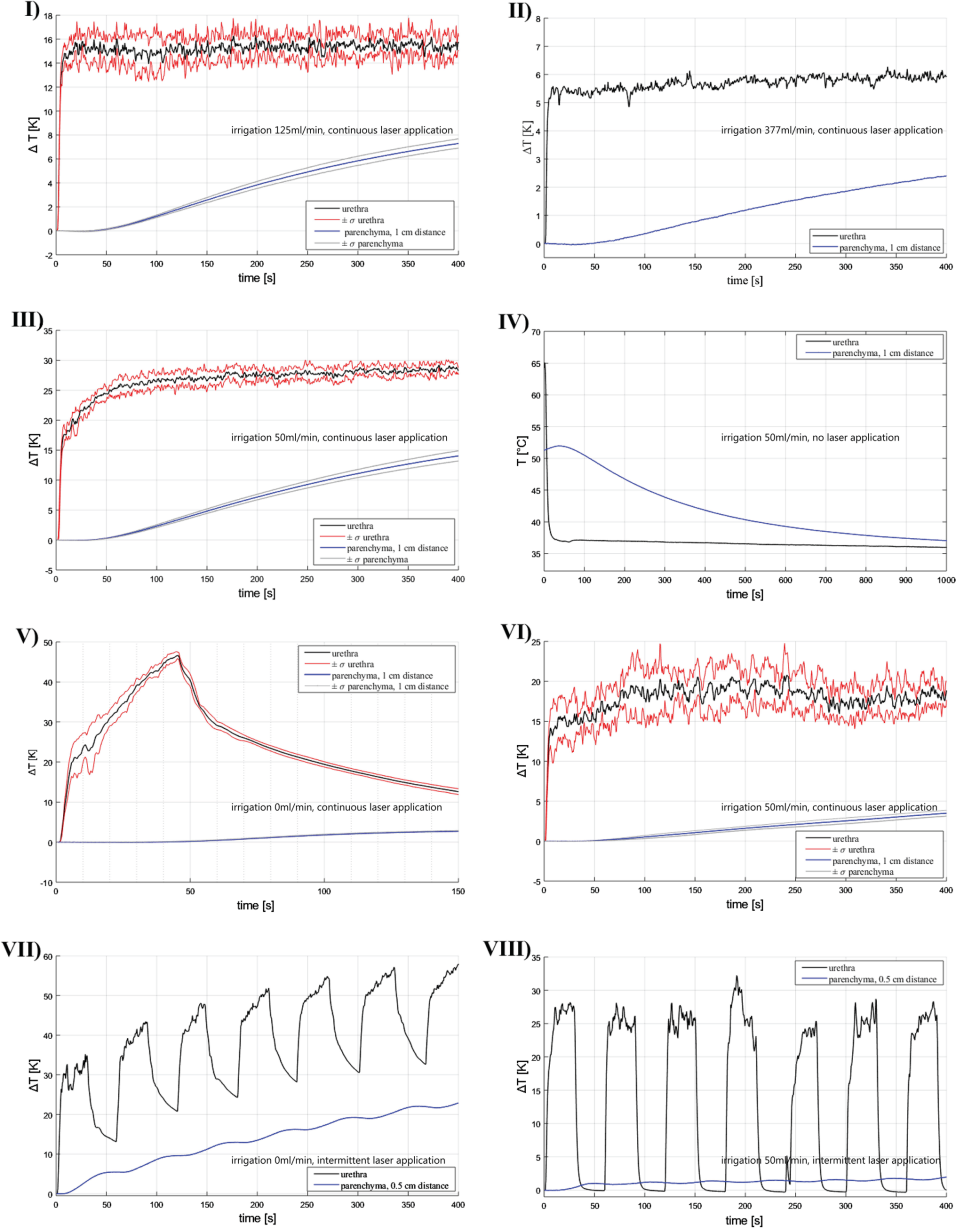
Results trial runs I-VIII

The experimental setup was based on a commercially available prostatic resection trainer (Samed GmbH, Dresden, Germany) placed in a 37 °C water bath (Fig. 1A), which was thermally homogenized by a hose pump (SP 04 L, Otto Huber GmbH, Böttingen, Germany). A hollowed-out central area simulates the urethral lumen and holds approximately 10 ml, where we placed a RigiFib 800 fibre (LISA laser products OHG, Katlenburg-Lindau, Germany), the inflow of irrigation regulated over a digital pump (Reglo Z, Cole Parmer, Chicago, Illinois, USA), and a thermocouple (type K, PICO Technology, Cambridgeshire, UK). A second thermocouple was inserted at a 0.5 cm/1 cm distance to the urethra and 3.5 cm deep into the prostatic parenchyma. Continuous laser application (Revolix^©^ Laser, LISA laser products OHG, Katlenburg-Lindau, Germany) up to 120 W was applied with several irrigation settings (37 °C) and trial durations of up to 14 min. Interrupted laser energy was applied to scenarios VII and VIII. To protect the parenchymal thermal couple from heated water coming directly from the urethra, we inserted a small aluminum barrier between the parenchymal couple and the urethra (Fig. 1B). Thermal measurements were recorded continuously and in real-time using MatLab (MatLab^®^ R2016b, The MathWorks, Inc., Natick, US). The irrigation outflow occurred passively by overflow. Measurements were repeated up to five times to balance out variations, standard deviations are displayed in the results section (Fig. 2).

We performed several measurement series with different irrigation / laser application settings which are displayed in Table 1. In trial runs VII and VIII, we simulated a surgical scenario in which 30 s laser application were followed by a 30 s halt in laser application intermittently.

**Table 1:** Measurement results of different irrigation and laser application settings

Trial run	Power [W]	Irrigation [ml/min]	Trial repeats	Trial duration [min]	Distance parenchymal couple	Laser mode
I	120	125	5	6:40	1 cm	Continuous application
II	120	377	1	6:40	1 cm	Continuous application
III	120	50	5	6:40	1 cm	Continuous application
IV^a^	0	50	1	16:40	1 cm	No laser application^a^
V	120	0	5	2:30	1 cm	Continuous application
VI	80	50	5	6:40	1 cm	Continuous application
VII	80	0	1	6:40	0.5 cm	Intermittent laser application 30 s followed by 30 s brake of application
VIII	80	50	1	6:40	0.5 cm	Intermittent laser application 30 s followed by 30 s brake of application

^a^No laser application, trial run after III to evaluate the temperature decrease

## Results

### General observations

In experimental runs I-VIII, we observed a rapid increase of urethral temperature. After several preliminary tests, we defined an irrigation of 125 ml/min as a first critical cut-off below which a potential harming urethral / parenchymal temperature can be reached.). In a clinical observation of several laser procedures at our department, we measured a maximum irrigation rate around 400 ml/min using a 26 Fr laser resectoscope (series shark, Richard Wolf GmbH, Knittlingen, Germany). At irrigation rates ≥ 377 ml/min (experimental run II), a urethral temperature increase of 6 K and a parenchymal increase of 2 K occurred both not critical regarding the CEM_43_ threshold (see Discussion). With regard to that, higher irrigation rates of 125 ml/min (I and II) lead to lower temperature increases compared to trial repetitions with an irrigation of 50 ml/min (III and VI) or no irrigation (V and VII). Measurement sessions I–V were performed with the maximum laser power of 120 W while VI to VIII were performed with a more realistic ThuLEP 80 W laser power.

### Specific observations

Series I simulated a continuous laser application of 120 W over 6:40 min. We observed a urethral ∆T of around 15 K in the steady state while there was a continuous parenchymal temperature increase of up to 7 K when laser application was stopped. In experimental run III, a reduced irrigation of 50 ml/min was used and we reached a urethral ∆T close to 30 K while the parenchyma reached a ∆T of 15 K. Trial run IV directly followed III and was performed to simulate urethral and parenchymal temperature decrease with a continuous irrigation of 50 ml/min. During trial IV no further laser application was performed. Notably, the y-axis in figure IV illustrates absolute values in degrees Celsius [°C] while all other illustrations illustrate ∆T in Kelvin [K]. There is instant urethral temperature normalization while the prostate parenchyma needs over 16 min to reach the baseline body temperature. Trial run V was performed to simulate a worst case scenario with no irrigation. We performed this trial only during 50 s because urethral temperature came close to boiling point (∆T ~ 45 K). Series VI to VIII simulated realistic surgical conditions in which we evaluated a laser power of 80 W. In trial VI, lower urethral and parenchyma temperature increases were reached compared to trial III where the same irrigation but the higher power of 120 W were evaluated. Experimental runs VII and VIII simulated more realistic surgical scenarios in which intermittent laser application (20 s laser application followed by 20 s brake) was evaluated instead of continuous. In trial VII no irrigation was applied, therefore a stepwise increase of urethral and parenchyma temperature occurred. In trial VIII, we showed that a low irrigation rate of 50 ml/min instantly reduces the urethral temperature peaks after laser application down to the baseline body temperature while there was no increase of the parenchymal temperature.

## Discussion

Current EAU guidelines recommend transurethral laser techniques as an alternative treatment option to TURP for prostates between 30 and 80 ml and as an equivalent treatment option (especially HoLEP) to open prostatectomy in glands > 80 ml [2]. With a weak recommendation, ThuVEP might be applied in patients under anticoagulant therapy [2]. While Ho:YAG and Tm:YAG lasers have almost the same wavelength of 2–2.1 µm, the Thulium laser´s continuous mode leads to a shorter tissue penetration of about 0.25 mm and may have a superior hemostasis [17]. While there is a lot of enthusiasm about laser-based LUTS treatment options, TURP remains the comparator for novel techniques and the gold standard in current guidelines [2]. A study evaluating the German health insurance database revealed that between 2008 and 2013 the prevalence of TURP decreased slightly from 83.4% (in total 14,935 procedures) to 78.7% (p < 0.05) while laser-based treatment alternatives increased from 7.4% to 12.9% (in total 1841 procedures, p < 0.05) and open prostatectomy remained stable at ~ 9% [18]. A steep learning curve—in particular in the enucleation techniques—retards their widespread adoption [19, 20]. The clinical safety profile of ThuVEP and ThuLEP has been demonstrated in a large case series and a RCT comparing ThuLEP and TURP [9, 10]. The efficacy and safety of ThuVARP vs. TURP was evidenced in a novel British RCT (“UNBLOCS trial”) with 410 participants in 2020, however, minor benefits were found in the TURP arm in terms of Qmax and prostate cancer detection [21]. Magistro et al. surmised in their Lancet comment on the UNBLOCS trial that a modest increased rate of nocturia in the ThuVARP arm might be caused by a median high energy of 148 kJ for a medium 35 g prostate size [21, 22]. A high cumulative energy during laser-based prostate surgery leads to increased temperatures of the irrigation fluid and lower urinary tract.

There is mounting evidence concerning the potentially harming thermal side effects of Ho:YAG and Tm:YAG lasers in in vitro investigations, which leads to the question whether there might be a relevant temperature increase of the irrigation fluid during Tm:YAG laser-based procedures [11–14]. The present work´s objective was to investigate the thermal effect of a 120 W Tm:YAG laser in a standardized in vitro prostate simulation setting on the surrounding irrigation fluid (urethra), the prostate parenchyma and deeper anatomical structures like the neurovascular bundles.

In all of our trial sessions, we reached a rapid temperature increase of the urethral irrigation fluid after continuous laser application. In trial runs I, III, V, and VI, we measured potentially harming temperatures in the urethra > 43 °C. The sensitivity of tissue on rising temperature is specific and defined in the cumulative equivalent at 43 °C (CEM_43_) at which potential thermal damage occurs [23]. In the literature, the canine urethra is defined as the most thermal sensitive tissue of the urinary tract characterized by a CEM_43_ ~ 1 min [24]—first tissue damage occurs at 43 °C, however, this is time-dependent. Notably, several measurement sessions simulated a worst case scenario with no irrigation (trial V) and with a low irrigation of 50 ml/min (III and VI) and 125 ml/min (I), all measured under continuous laser application over 400 s. On one hand, the temperature rose instantly after a few seconds to a steady state, while on the other hand, continuous laser application is an unrealistic surgical scenario even for experienced surgeons. With a normal to high irrigation rate of 377 ml/min (trial run II), the urethral temperature rise was ~ 5 K in the steady state under continuous laser application. From a starting 37 °C temperature, this leads to 42 °C in the urethra. Consequently, the measured urethral temperature in II might be harmless.

In trial runs VII and VIII, we simulated two realistic surgical scenarios. These trial runs were conducted with thermocouples inserted at a 0.5 cm distance to measure thermal exposition of anatomical structures that might be very close to the prostate (e.g., neurovascular bundle). Herein, 30 s of laser application were followed by a halt of 30 s. In VII, no irrigation was applied and we measured a stepwise increase of the urethral temperature. In clinical practice, this scenario plays a minor role because surgeons will usually stop after a few seconds faced to no irrigation because there will be no proper endoscopic vision. In trial run VIII with an irrigation of only 50 ml/min, we observed a fast urethral temperature increase during laser application followed by an equally fast decrease after the laser is paused. In this more realistic surgical scenario no significant temperature increases of the prostate parenchyma were observed.

Regarding the parenchymal temperature developments, we observed interesting results in all trial runs (blue curves, Fig. 2). In all measurement sessions with laser application, a continuous increase of the parenchyma was observed. In trial run III with a high laser power of 120 W and low irrigation of 50 ml/min a ∆T of 15 K was reached. This might be a potentially harming temperature, however, in the parenchyma this harming effect plays no role because the procedure´s aim is the adenoma´s removal. However, we placed the thermocouple 1 cm away from the urethra and the source of energy. In a laser application scenario near the prostate capsule, structures behind the prostate such as the neurovascular bundles might be exposed to heat and damaged. In trial run IV, we observed further temperature development (in °C) after laser application from trial run III followed by laser deactivation and further irrigation with 50 ml/min. Interestingly, it took approximately 8 min for the temperature to decrease to 40 °C. Notably, we could not simulate blood circulation in our model—although we circulated the water bath by a hose pump—which might lead to a faster temperature decrease. However, this natural cooling effect could not be demonstrated by Aldoukhi et al. in a in vivo model evaluation of the thermal effect of Ho:YAG laser lithotripsy in the porcine renal pelvis [15]. All in all, we showed that it is possible to increase the temperature of the prostate parenchyma and deeper anatomical structures to potentially harming levels.

This aspect is more relevant for laser powers exceeding 120 W. As reported in the literature, some surgeons use laser powers of up to 200 W for Thulium-based prostate surgery [25]. The same retrospective study postulates no raised complication rate in this high power ThuLEP case series [25]. Nevertheless, as shown in the present study and multiple in vitro settings, higher laser powers are linked to higher temperature developments [11–16, 26].

Other groups go further and postulate a correlation between high temperatures in the urinary tract during laser-based procedures and postoperative symptoms like nocturia and urgency [27]. The same group conducted a RCT with 100 patients in 2014 comparing diode laser vaporisation of the prostate with irrigation at room temperature versus vaporisation with cooled irrigation at 4 °C [27]. Herein, Qmax showed no differences at 1-month follow-up while IPSS, QoL, post residual voiding volume, transient urgency, and stress incontinence showed significant advantages in the study arm with cooled irrigation at 1-month follow-up [27]. However, at 3- and 12-month follow-up no further significant differences of the above mentioned outcome parameters could be measured [27]. Further evaluations, in particular multicenter trials, are necessary to elucidate this hypothesis.

The present study has several limitations. In session V, we may have had a systematic error with the thermocouple in the prostate parenchyma because the measured temperature increase was not comparable to all other trial runs. Additionally, this error was balanced out because we had a small standard deviation over the five repetitions of trial run V (see results section). In our study, warmed irrigation was used. However irrigation on room temperature may widen the safe corridor in regard to resulting harming temperatures. This was not further investigated in the present study. Our study was limited to a Tm:YAG laser as the only energy source. However, other energy sources like the Ho:YAG laser may theoretically have an impact to the thermal effects. But we postulate that the energy source plays a minor role at the same power settings.

Furthermore, the in vitro setup and thermocouple placement could not entirely simulate the prostate anatomy, surrounding pelvis, and blood circulation all influencing the temperature development.

## Conclusions

Tm:YAG laser-based LUTS treatment is an alternative option to TURP, HoLEP, open prostatectomy as stated in present EAU guidelines. During Tm:YAG laser application, the surrounding irrigation fluid might be heated. In the present in vitro study design, we showed that potentially harming temperature values could be reached particularly working with high laser power and at low irrigation. The heat generation can also be dissipated to the deeper prostate parenchyma, potentially warming the neurovascular bundles. Further clinical studies with intracorporal temperature measurement are necessary to explore this potentially harming side effect in greater detail.

## Abbreviations

K: Kelvin
Tm:YAG: Thulium:yttrium–aluminium-garnet laser
